# Understanding Melasma-How Can Pharmacology and Cosmetology Procedures and Prevention Help to Achieve Optimal Treatment Results? A Narrative Review

**DOI:** 10.3390/ijerph191912084

**Published:** 2022-09-24

**Authors:** Zuzanna Piętowska, Danuta Nowicka, Jacek C. Szepietowski

**Affiliations:** 1 Department of Dermatology, Venereology and Allergology, Wrocław Medical University, 50-368 Wrocław, Poland; 2 Faculty of Physiotherapy, Wroclaw University of Health and Sport Sciences, 51-612 Wrocław, Poland

**Keywords:** melasma, hyperpigmentation, melasma pathogenesis, melasma treatment, laser therapy, photoaging, chemical peels, hydroquinone

## Abstract

Melasma is a chronic skin condition that involves the overproduction of melanin in areas exposed to ultraviolet radiation. Melasma treatment is long-term and complicated with recurrence and resistance to treatment. The pathogenesis of melasma is highly complex with multiple pathologies occurring outside of the skin pigment cells. It includes photoaging, excessive melanogenesis, an increased number of mast cells, increased vascularization, and basement membrane damage. In addition, skin lesions related to melasma and their surrounding skin have nearly 300 genes differentially expressed from healthy skin. Traditionally, melasma was treated with topical agents, including hydroquinone, tretinoin, glucocorticosteroids and various formulations; however, the current approach includes the topical application of a variety of substances, chemical peels, laser and light treatments, mesotherapy, microneedling and/or the use of systemic therapy. The treatment plan for patients with melasma begins with the elimination of risk factors, strict protection against ultraviolet radiation, and the topical use of lightening agents. Hyperpigmentation treatment alone can be ineffective unless combined with regenerative methods and photoprotection. In this review, we show that in-depth knowledge associated with proper communication and the establishment of a relationship with the patient help to achieve good adherence and compliance in this long-term, time-consuming and difficult procedure.

## 1. Introduction

Melasma (chloasma) is a chronic acquired skin condition involving the overproduction of melanin in areas exposed to ultraviolet radiation. Melasma usually appears as symmetrically located irregular macules and patches that are light brown to dark brown in color, developing mainly on the face, and much less often on the neck and forearms [1,2]. The pathogenesis of melasma is not fully elucidated; however, it is known that it occurs much more often among women with darker skin complexions (according to Fitzpatrick skin phototypes III–V) in the third and fourth decade of life. Hormonal factors such as oral contraceptives, pregnancy, genetic factors, chronic inflammation of the skin and prolonged exposure to solar radiation remarkably affect the etiopathogenesis and development of melasma [3,4,5,6]. Much less frequently, melasma is caused by the use of photosensitizing substances, thyroid diseases, hepatopathies, ovarian tumors, the consumption of certain foods, parasitic infestations, and even increased stress [7,8]. Several studies showed a very varied prevalence of melasma, ranging from 1% in the general population to up to 9–50% in populations at risk [9]. Some diseases tend to develop more frequently with melasma, probably due to the shared pathomechanism; however, there are only a few reports in the literature on melasma comorbidities. Menstrual cycle irregularities associated with polycystic ovarian syndrome and insulin resistance were reported to be diagnosed more often in women with melasma. Similar findings were reported for thyroid dysfunction and depression which may also be driven by hormonal imbalance [10]. Psychiatric conditions such as depressive and stress disorders are diagnosed in 76% of patients with melasma [11].

**Figure 1:**
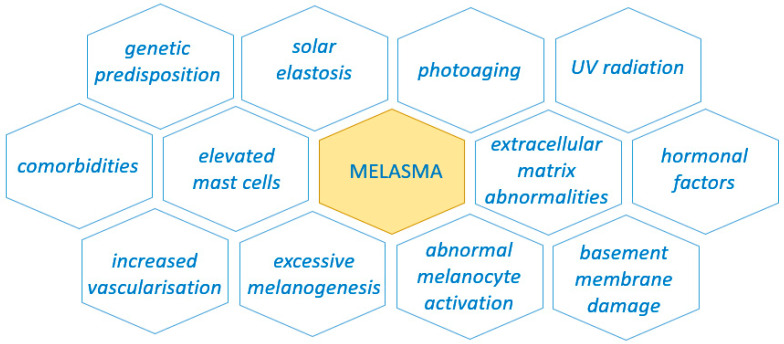
Factors contributing to the development of melasma.

The complexity of factors contributing to the development of melasma is shown in Figure 1.

The term melasma comes from the Greek word “mélas” which means black and refers to its clinical picture. The first descriptions of this disease can be found in the ancient medical literature (470–360 BC) written by Hippocrates. He used this term to describe a series of skin pigment disorders that deteriorated under the influence of solar radiation, high and low temperature and inflammation of the skin [12].

This disease has a significant impact on emotional and psychological wellbeing, and strongly deteriorates the quality of life of the patients. The disease is often neglected, and is unfortunately seen as merely a cosmetic defect, leading to underdiagnosis and incorrect treatment. Affected patients report feelings of embarrassment, frustration and uncertainty [13]. In 2019, Dabas et al. [14] examined the frequency of occurrence of psychological disturbances in people with pigmentary disorders, for which the results clearly indicated an increased incidence of anxiety and depression in patients with melasma. Moreover, disease-specific health-related quality of life (HRQOL) tools have been developed and validated. They identify the areas of the patient’s life most affected by the occurrence of this dermatosis, as well as its impact on the general level of functioning in correlation with the severity of the disease. Such tools include the Melasma Quality of Life scale (MELASQOL) and multidimensional questionnaire for evaluating quality of life in melasma (HRQ-melasma) [15,16,17,18,19,20]. The Melasma Area and Severity Index (MASI) is a reliable measure of melasma severity. The area affected by the disease and the degree of progression of the lesions is sufficient to accurately measure the severity of this disease.

Treatment of melasma is long-term and complicated, as it is often resistant to therapy or recurs despite constant appropriate treatment; therefore, it seems very important to develop an understanding of pathogenetic pathways in order to effectively treat and prevent this disease.

## 2. Pathological Molecular Mechanisms

The pathogenesis of melasma is highly complex; however, in recent years, numerous studies have shed a completely new light on it. Initially, pathologies were thought to be only related to melanocytes; however, we now know that the disturbances extend far beyond the skin pigment cells, as they also include the interaction of keratinocytes, abnormal melanocyte activation, aggregation of melanin and melanosomes in the epidermis and dermis, an increased number of mast cells, increased vascularization, basal membrane damage, skin extracellular matrix abnormalities and photoaging (solar elastosis) [3].

The patients show complex clinical and histological characteristics, suggesting the involvement of multiple pathogenetic pathways [21]. The analysis of the transcriptional activity of melasma-related skin lesions showed that nearly 300 genes are differentially expressed in skin lesions and in the surrounding skin, which only emphasizes the complexity of etiopathogenesis [22].

### 2.1. Solar Elastosis, Photoaging, Extracellular Matrix Abnormalities

Solar elastosis (actinic elastosis) is a pathological condition that involves the accumulation of elastic fibers and the degeneration of elastic tissue after excessive exposure to solar radiation (photoaging of the skin). UVA radiation penetrating deep into the layers of the dermis plays a special role in the development of this pathological process [23]. Overall, 93% of patients with melasma had a moderate to severe degree of solar elastosis [24]. A significantly higher degree of disturbances in the extracellular matrix, and thus solar elastosis, was observed in skin affected by melasma, compared to the surrounding skin without pathological changes [25,26]. Kwon et al. [4] strongly suggest that melasma should not only be perceived as a disease of melanocytes, but also as a pathological condition closely related to photoaging of the skin.

### 2.2. Abnormal Activation of Melanocytes and Excessive Melanogenesis

Melasma is characterized by the presence of biologically hyperactive melanocytes. One melanocyte maintains a connection with 36 keratinocytes to form an epidermal melanin unit [27]. In addition to the above-mentioned UVA, UVB also has a significant impact on the formation of hyperpigmentation, which stimulates keratinocytes to produce growth factors, including stem cell factor (SCF), basic fibroblast growth factor (bFGF), interleukin 1 (IL-1), endothelin 1 (EDN1), inducible nitric oxide synthase (iNOS), α-melanotropin (α-MSH), adrenocorticotropin (ACTH) and prostaglandin E2 (PGE2, dinoprostone) [28,29,30,31,32]. These products, directly and indirectly, induce melanogenesis; however, the effect on melanocyte proliferation is further discussed in [33,34]. Ultraviolet radiation, as well as paracrine, autocrine, and hormonal factors regulate melanogenesis through numerous signaling pathways including cAMP/PKA/CREB/MITF, NO/cGMP/PKG, and PLC/DAG/ PKCβ [35].

It is an indisputable fact that the effect of UV on the upregulation of MSH receptors, also known as melanocortin 1 receptors (MC1R), results in increased hormone binding, and thus increased melanin production [36]. Furthermore, in response to UV, proopiomelanocortin (POMC) secreted by the pituitary gland undergoes post-translational processing, during which it is cleaved, resulting in MSH and ACTH, which bind and activate the MC1R receptor and indirectly activate tyrosinase [2].

The production of endogenous 1,2-diacylglycerols (DAG) with the activation of protein kinase C (PKC) and nitric oxide (NO) formation along with the synthesis of cyclic guanylate monophosphate (cGMP) serves as another signaling pathway. This pathway, as a result of ultraviolet radiation, leads directly to the stimulation of melanogenesis [21].

Skin fibroblasts also secrete Wnt signaling modulators, which stimulate melanogenesis and melanosome transfer, in melasma gene upregulation for these proteins [22,37]. Promelanogenic growth factors such as keratinocyte growth factor (KGF) or hepatocyte growth factor (HGF) and SCF are produced by fibroblasts isolated from photo-damaged skin. These observations suggest that fibroblasts may also play an important role in the multifaceted pathogenesis of melasma [38].

Another important factor in the development of excessive melanogenesis is UV-induced cyclooxygenase-2 (COX-2). Kim et al. [39] investigated how silencing COX-2 expression would affect melanin production and the expression of melanogenic factors. They found that cells transfected with COX-2 small interfering RNA (siRNA) to silence COX-2 expression showed a reduced production and activity of tyrosinase, tyrosinase-related protein 1 (TRP1), TRP2, glycoprotein 100, and MITF. Moreover, these cells showed a decreased production of melanin induced by α-MSH, which makes COX-2 inhibitors an important therapeutic option in this disease.

Despite all-face exposure to sunlight, melasma typically occurs only in specific areas that are rich in sebaceous glands (cheeks, forehead, and upper lip) [40]. This is most likely related to the ability to synthesize pro-inflammatory cytokines and growth factors regulating melanogenesis by the sebaceous glands. Cultivation of the sebocyte cell line together with human melanocytes induces melanogenesis, which shows that certain factors secreted from sebocytes may play an important role in this process [41].

Significantly increased superoxide dismutase (SOD) activity and decreased glutathione levels in patients with this disease indicate the presence of elevated oxidative stress in melasma [42].

### 2.3. Increased Number of Mast Cell

The number of mast cells in skin affected by melasma is overwhelmingly higher than in healthy skin [43]. Mastocytes are present in areas of the skin with increased elastosis and the elastin content in the skin exposed to ultraviolet radiation correlates with the number of mast cells. Research has shown that the development of elastic fibers in solar elastosis is stimulated by mast cells directly by stimulation of fibroblasts or indirectly by other types of cells [25,26,44]. Released from mast cells after ultraviolet irradiation, tryptase, granzyme B and activated extracellular matrix metalloproteinases (MMP) participate in the degradation of the basement membrane by decomposing collagen IV [45,46,47]. Histamine, secreted from cells under the influence of ultraviolet radiation, binds to the histamine 2 receptor, activates the tyrosinase pathway and induces melanogenesis, which may explain the direct relationship between inflammation associated with solar radiation and the occurrence of discoloration [48,49].

### 2.4. Increased Vascularization

The formation of new vessels in healthy skin is negligible under normal conditions, but can increase in certain pathological conditions such as chronic inflammation or after prolonged exposure to ultraviolet rays. Research has shown that skin with melasma is vascularized to a greater extent than skin without pathological changes [50]. This is due to an increase in the number of mast cells, which induce vascular proliferation by secreting bFGF, vascular endothelial growth factor (VEGF) and transforming growth factor—β (TGF-β). VEGF is also upregulated in keratinocytes, and functional VEGF receptors are found on melanocytes. This factor stimulates the production and release of the metabolites of arachidonic acid and phospholipase A2 [50,51]. Moreover, VEGF receptors are also found on vascular endothelial cells that stimulate melanogenesis by producing EDN1, thereby stimulating MITF phosphorylation and increasing tyrosinase levels. Researchers have shown a statistically significant relationship between the number of vessels and hyperpigmentation in melasma [42]. Additionally, angiogenetic factor levels increase with the size, density and diameter of vessels in the affected skin [3,4,46]. Telangiectatic erythema is another feature that distinguishes the skin with melasma, making it another therapeutic target.

### 2.5. Basement Membrane Damage

Ultraviolet radiation causes the release and activation of MMP2 and MMP9 from mast cells, which is associated with the destruction of collagen IV and VI in the basement membrane [3]. Damage to the basement membrane also allows melanocytes and melanin molecules to migrate into the dermis, which contributes to the persistent and recurrent nature of melasma. For this reason, an important problem is the appropriate selection of therapy and careful use of laser techniques, which, if used inappropriately, may worsen the course of the disease.

### 2.6. Genetic Component

Melasma is common in people with darker skin in whom familial predisposition (genetic component) is considered an important risk factor. This skin condition was reported in twin sisters while it did not develop in the other not-twin sister, indicating susceptibility to this disease [52]. An international survey conducted among women treated for melasma showed that 48% of respondents confirmed a family history of melasma with 97% of cases occurring in a first-degree relative [53]. A Brazilian study found the familial occurrence of melasma in 56.3% of patients with melasma [54]. Microarray analysis of hyperpigmented skin from patients with melasma showed downregulation of the H19 gene which was not seen in the skin of patients not affected by melasma [55]. A transcriptomic study found that 279 genes were differentially expressed in lesional and perilesional skin. Bioinformatics analysis led to the conclusion of the upregulation of genes involved in melanin production (TYR, TYRP1, DCT, and SILV) and downregulation of genes involved in the lipid metabolism (PPARA, arachidonate 15-lipoxygenase, type B, diacylglycerol O-acyltransferase2-like 3, and PPAR gamma coactivator 1 alpha) [22]. Although many attempts have been undertaken to identify a Mendelian pattern of segregation, it is still to be discovered.

## 3. Management

Therapy of melasma appears to be extremely difficult due to its complex, multifactorial and multi-level etiology, treatment resistance and high relapse rate. Previously, therapeutic management was based on topical treatment, which did not eliminate relapses and exacerbations of the disease. When treating melasma, hormonal imbalances must be taken into consideration. The essential factor is photoprotection, and another important step is the use of a combined multimodal approach with appropriate maintenance therapy. The main principles of the therapy of discoloration in melasma are: inhibition of melanin synthesis pathways, drop of melanosome transfer from melanocytes to keratinocytes and promotion of melanin removal pathways. An ideal therapeutic approach should involve various pathogenetic mechanisms to obtain the best possible results. The basis is year-round photoprotection with broadband filters with a very high protection factor (SPF 50+ and PPD+++ or PPD++++) [56], as well as protection against sunlight in the form of protective clothing and avoiding peak exposure to radiation. Traditionally, melasma has been treated with topical agents, including hydroquinone (HQ) (tyrosinase inhibitor), tretinoin, glucocorticosteroids and various formulations. HQ has been the first-line therapy for a long time, but concerns about its side effects have prompted the use of potentially safer alternatives and the withdrawal of HQ in many countries. The current approach includes the topical application of a variety of substances, chemical peels, laser and light treatments, mesotherapy and microneedling or the use of systemic therapy [21]. The newest, innovative methods of treating skin diseases and reducing signs of aging that are also used in melasma include stem cells and their products [57]. Stem cell factor (SCF) has been found to show increased expression in hyperpigmentation in melasma, lentigo or freckles. It can be used as a target to develop new treatments for hyperpigmentation via the inhibition of SCF [58].

### 3.1. Pharmacological Treatment

Topical medications are still the first choice for the treatment of hyperpigmentation diseases, with HQ being the gold standard for treating melasma in many countries. It is an organic chemical compound from the phenol group, which remains the most popular and one of the most effective anti-melanogenic agents, inhibiting the conversion of 1-3,4-dihydroxyphenylalanine to melanin by the competitive inhibition of tyrosinase [59]. Studies showed that the triple combination cream of 4% HQ, 0.05% tretinoin and 0.01% fluocinolone acetonide was slightly more effective than 4% HQ alone or in dual combination; thus, it is the only drug containing HQ approved by the U.S. Food and Drug Administration (FDA) [60,61,62,63,64]. The safety issues related to HQ are unfortunately still controversial and unclear, which is why the European Commission has banned the substance due to its possible complications, such as exogenous ochronosis, permanent depigmentation or even the potential risk of cancer due to HQ metabolites (p-benzoquinones) [65,66]. This led to a further search for substitutes with a similar efficacy and a lower risk of side effects.

Table 1 summarizes some possible topical pharmacological therapies for melasma and the pathogenic pathways they affect. Substances recently used in melasma such as 4-n-butylresorcinol, tranexamic acid (TA), cysteamine, niacinamide, pycnogenol or thiamidol seem to be a promising alternative therapy with satisfactory results; however, further randomized placebo-controlled trials involving large groups of patients are necessary to confirm the effectiveness of these substances [67,68].

Tretinoin 0.05–0.1% reduces skin pigmentation by inhibiting the transcription of tyrosinase and interrupting melanin synthesis. Retinoids also support the metabolism and turnover of keratinocytes, reducing melanosome transfer and accelerating melanin loss, as well as facilitating the transepidermal penetration of other topical medications [69,70]. Although tretinoin may be effective in reducing discoloration, it usually takes at least 24 weeks to achieve clinical improvement, and this therapy may be associated with secondary hyperpigmentation to retinoid-induced irritation [71,72]. Other retinoids have also been used to treat melasma, including adapalene, tazarotene and topical isotretinoin [73,74].

Oral tranexamic acid should also be taken into account in the treatment of melasma, which even in low doses (e.g., 500 mg/day) in systematic reviews is presented as an effective and safe drug in monotherapy or in combination with routine treatment methods [75,76,77]. TA is an antifibrinolytic agent that affects hyperpigmentation through several mechanisms, including the inhibition of epidermal melanocyte tyrosinase activity, preventing plasminogen binding to keratinocytes and reducing α-MSH [78]. It acts as a plasmin inhibitor by reducing the concentration of arachidonic acid, prostaglandins and leukotrienes in keratinocytes. TA also inhibits angiogenesis through the suppression of VEGF and EDN1. This substance is one of the few methods of treating neovascularization in this disease. The most common side effects of oral TA reported in studies include oligomenorrhea, gastrointestinal discomfort, headache and transient skin irritation [79,80,81]. According to these studies, this substance does not increase the risk of thromboembolism; however, appropriate screening for personal and familial risk factors, qualification and physical examination of patients before initiating treatment is an extremely important element in avoiding this type of complication [76,82]. Further research is needed to determine the optimal dose and treatment regimen and possible combination therapy with TA. It is worth adding that this substance can also be used topically, in the form of an injection as mesotherapy or in microneedling [2], however the oral prescription of TA showed the best clinical results.

Another promising substance is thiamidol, a tyrosinase inhibitor, which is effective in preventing pigmentation changes caused by UVB radiation [83]. Lima et al. compared a cream with 0.2% thiamidol to one with 4% HQ in a blinded, randomized clinical trial, the results of which were very surprising as the improvement after 90 days of using the above-mentioned preparations did not differ in both groups [84]. Thiamidol can be considered an appropriate therapeutic option for patients with melasma who experience poor tolerance to treatment or treatment failure with HQ [85,86].

However, other formulations routinely administered orally in studies applied to the skin were antioxidants such as ascorbic acid and zinc [87,88]. Ascorbic acid is an inhibitor of melanogenesis through its antioxidant effect and interaction with copper ions at the active site of tyrosinase [89]. With the topical application of both vitamin C and zinc, study patients saw an improvement in skin lesions with relatively minor side effects [90,91]. Using natural antioxidants was reported to bring positive results as well. Substances such as Korean red ginseng, plant extracts including orchid extracts, and parsley showed good effectiveness and tolerability; thus, they can be considered as an adjunct treatment for melasma [92,93,94].

These topically applied substances can be included as an additional step in the melasma therapy and skin care plan of patients with melasma; however, it should be remembered that they have a small potential for action, which is much less than HQ.

In the oral treatment of this disease, it is worth considering supplementation with systemically applied antioxidants to reduce oxidative stress, e.g., pycnogenol. This substance is a standardized plant extract from the pine bark of Pinus pinaster. The extract consists of procyanidins, polyphenols, phenolic and cinnamic acids and their glycosides. Its main advantage is its high bioavailability, the synergistic action of the ingredients and the low incidence of side effects when administered orally. In studies, patients with melasma reported a reduction in hyperpigmentation after only a month of treatment with pycnogenol [87,95,96].

Another target pathway in the treatment of melasma is the interaction between keratinocytes and melanocytes. Several cosmeceutical agents are available, such as niacinamide and soy, which bind to the protease-activated receptor 2 (PAR-2) and stop the transfer of melanosomes to surrounding keratinocytes [89]. Serine protease inhibitors, lectins and neoglycoproteins also influence this process [65].

Recent advances in the pharmacological therapy of melasma include very specific measures targeting different links in the pathogenetic pathways in melasma. Therapeutic alternatives to traditional topical agents such as siRNA agents have been explored [97]. The use of MITF-siRNA as a transdermal target peptide inhibits the tyrosinase pathway without major side effects. This novel option also shows promise in the treatment of melanoma and is safe enough for daily use at home.

Another very interesting and promising finding is the use of metformin on the skin; this anti-diabetic drug works by lowering the level of cAMP, thus reducing the melanin content in melanocytes by inhibiting further synthesis pathways [98]. Researchers also applied another oral drug, a proton pump inhibitor (PPI), omeprazole, to the discolored skin, which can also inhibit the formation of pigment. PPIs are believed to interfere with ATP7A by blocking tyrosinase copper uptake, leading to its degradation and thus reducing melanogenesis [99].

Summarizing, monotherapy with topical HQ or combined therapy with retinoids and corticosteroids has the greatest evidence of efficacy in the treatment of melasma. However, topical treatment can be unsatisfactory for the patient due to the slow improvement, frequent relapses, or side effects such as skin irritation, erythema and post-inflammatory hyperpigmentation (PIH) [100,101] or the unavailability of certain therapeutic substances in the European Union. For these reasons, both medics in everyday clinical practice and patients are looking for alternative, effective and safe methods to treat melasma.

**Table 1:** Summary of topical therapies in melasma and the pathogenetic pathways they affect.

Name of the Substance	Method of Application	Pathogenetic Mechanism	Side Effects	Reference
4-n-butylresorcinol (Rucinol)	Topical	Tyrosinase inhibitor Inhibitor TRP-1	Not reported	Mohan et al. [102] Kwon et al. [103] Sarkar et al. [87] Huh et al. [104] Khemis et al. [105]
Arbutin	Topical	Tyrosinase inhibitor Inhibitor DHICA Inhibition of melanosome maturation	Not reported	Sarkar et al. [89] Morag et al. [106]
Ascorbic acid	Topical Oral	Decreasing the dopaquinone and DHICA oxidation Antioxidant Tyrosinase inhibitor via copper ions Photoprotective effect	Skin irritation	Espinal-Perez et al. [107] Huh et al. [108]
Azelaic acid	Topical	Tyrosinase inhibitor Melanocyte inhibitor	Skin irritation	Baliña and Graupe [109] Farshi [110] Mazurek and Pierzchała [111] Verallo-Rowell et al. [112]
Calcineurin inhibitors	Topical	Induction of mast cell apoptosis Anti-inflammatory effect	Burning sensation	Kirsch et al. [113]
Cysteamine	Topical	Tyrosinase inhibitor Peroxidase inhibitor Iron and copper chelator Increase in intracellular glutathione	Skin irritation Unpleasant odor	Mansouri et al. [114]
Dioic acid	Topical	Intranuclear PPAR receptor agonist Reduction in melanosome transfer	Skin irritation	Tirado-Sánchez et al. [115]
Flutamide and other anti-hormonal substances (estrogen antagonists)	Topical	Anti-hormonal effect (anti-androgenic, anti-estrogenic) Reduction in the concentration of α-MSH and cAMP	Not reported	Adalatkhah et al. [116] Cohen [117]
Glycolic acid	Topical	Tyrosinase inhibitor Increase keratinocyte turnover	Skin irritation	Sarkar et al. [118] Sahu and Dayal [119] Chaudhary and Dayal [120] Lim [121] Khunger et al. [122] Borelli and Fischer [123] Hurley et al. [124] Ilknur et al. [125] Kumari and Thappa [126] Faghihi et al. [127] Erbil et al. [128] Dayal et al. [129]
Hydroquinone	Topical	Tyrosinase inhibitor Peroxidase inhibitor Melanocyte inhibitor Destruction of melanocyte cell membranes	Skin irritation Nail discoloration Colloid milia Transient skin discoloration Exogenous ochronosis	Tse [59] Ennes et al. [130] Sanchez et al. [33] Baliña and Graupe [109] Verallo-Rowell et al. [112] Farshi [110] Guevara and Pandya [131]
Kojic acid	Topical	Tyrosinase inhibitor	Skin irritation	Monteiro et al. [132] Deo et al. [133]
Linoleic, α-linolenic and oleic acid	Topical	Photoprotective effect Increase keratinocyte turnover	Not reported	Ando et al. [134]
Metformin	Topical	Inhibition of cAMP accumulation, CREB phosphorylation and MITF accumulation	Not reported	Lehraiki et al. [98]
Methimazole	Topical	Peroxidase inhibitor Melanocyte inhibitor	Systemic absorption was not observed	Kasraee et al. [135] Gheisari et al. [136]
Niacinamide	Topical	Melanosome transfer inhibition Melanocyte inhibitor Reduction in solar elastosis Anti-inflammatory effect Anti-aging effect (stimulation of ceramide production) PAR-2 inhibitor	Skin irritation	Navarrete-Solis et al. [137]
Photobiomodulation	Topical	Melanocyte inhibitor (by tyrosinase, TRP-1, MITF) Modulation of p53 expression	Not observed	Barolet [138]
Proton pump inhibitors	Topical	Blocking ATP4A and ATP7A Increased degradation of tyrosinase	Not reported	Matsui et al. [99]
Pycnogenol	Topical Oral	Antioxidant Anti-inflammatory effect	Not reported	Sarkar et al. [89] Lima et al. [139]
Retinoids	Topical	Inhibition of UVB-stimulated keratinocytes Inhibition of tyrosinase transcription Reduction in melanosome transfer Increase keratinocyte turnover	Skin irritation	Griffiths et al. [72] Kang et al. [140] Shroot et al. [73] Leenutaphong et al. [74] Khunger et al. [122] Ghersetich et al. [141] Kimbrough-Green et al. [142] Truchuelo et al. [143] Dogra et el. [144]
siRNA	Topical	Tyrosinase inhibitor MITF Inhibitor	Not reported	Yi et al. [97]
Steroids	Topical Intradermal	Inhibition of recruitment and maturation of mast cells Anti-inflammatory effect	Skin atrophy Telangiectasia Steroid acne	Kanwar et al. [145] Nassar et al. [146] Eshghi et al. [147]
Silymarin	Topical	Antioxidant Anti-inflammatory effect	Not reported	Nofal et al. [148] Altaei [149]
Thiamidol	Topical	Tyrosinase inhibitor	Not reported	Arrowitz et al. [85] Roggenkamp et al. [86] Lima et al. [84] Philipp-Dormston et al. [150]
Tranexamic acid	Oral Topical Intradermal	Tyrosinase inhibitor and melanocytes inhibitor Mast cell downregulation Plasmin inhibitor (reducing the amount of arachidonic acid and α-MSH) Reduction in solar elastosis Lowering VEGF and endothelin 1	Oligomenorrhea Gastrointestinal disorders Skin irritation Headache Thromboembolic complications	Ebrahim et al. [151] Bala et al. [76] Janney et al. [152] Wu et al. [80] Atefi et al. [153] Lueangarun et al. [154] Banihashemi et al. [155] Laothaworn and Juntongjin [156] Xu et al. [157] Saki et al. [158] Tehranchinia et al. [159] Steiner et al. [160] Budamakuntla et al. [161] Sharma et al. [162]
Trichloroacetic acid	Topical	Increase keratinocyte turnover	Skin irritation	Soliman et al. [163] Abdel-Majid et al. [164] Sahu and Dayal [119] Abdel-Meguid et al. [165] Murtaza et al. [166]
Triple combination cream	Topical	Tyrosinase inhibitor Peroxidase and melanocyte inhibitor Destruction of melanocyte cell membranes Inhibition of UVB-stimulated keratinocytes Inhibition of tyrosinase transcription Reduction in melanosome transfer Increase keratinocyte turnover Inhibition of recruitment and maturation of mast cells Anti-inflammatory effect	Skin irritation Burning Dryness Pruritus	Taylor et al. [62] Torok et al. [167] Ferreira et al. [60] Chan et al. [64] Gong et al. [63] Arellano et al. [168] Grimes et al. [169]

### 3.2. Chemical Peels

Chemical peels are often used in the treatment of melasma; their main advantage is the range of substances and the depth of their penetration, which can be properly adjusted depending on the patient’s needs. In melasma, we use superficial or medium-depth peels, mainly synergistically with local treatment, other in-office treatments and photoprotection. The main disadvantage of this method is the occurrence of complications, such as PIH, most often occurring in patients of Asian origin with Fitzpatrick III–IV skin type [87]. Deep peels are generally not used in melasma because they are associated with possible complications, including PIH and hyperpigmentation, scarring, secondary infections of damaged skin, persistent post-inflammatory erythema, milia formation or abnormal healing.

Chemical substances used in peels, alpha and beta hydroxy acids such as glycolic, salicylic, lipohydroxy, pyruvic, lactic, almond, Jessner’s formula, azelaic and trichloroacetic acid (TCA) have been present in medicine for decades and have been extensively researched for their effectiveness in treating resistant melasma [111,118,119,120,123,163,164,170,171,172,173,174,175].

Sarkar et al. [118] compared the therapeutic efficacy and tolerance of 35% glycolic acid (GA) (group A) to salicylic acid in combination with mandelic acid (20% salicylic acid and 10% mandelic acid) (group B) and to phytic acid (group C) in Indian melasma patients. Each group was prepared for 4 weeks before the procedure with 4% HQ and 0.05% tretinoin. The chemical peel was performed every 14 days until the 12th week of the study. Improvement was seen in all 3 groups, but was statistically significant in group A compared to group C, and in group B compared to group C, but there was no statistically significant difference between groups A and B.

Sahu et al. [119] examined the differences in the effectiveness of peelings using 15% TCA, 30% GA and 92% lactic acid. They showed that 15% of TCA is as effective as 30% of GA, while both exceeded the effectiveness of 92% of lactic acid. However, GA was better tolerated by the patients; therefore, according to the researchers, any of these peels can be used in clinical practice after taking into account the patient’s profile. The authors suggest that the 30% GA peel is the best of the three peels; however, for aging, thin and sensitive skin, lactic acid may be preferred.

Another substance that has recently been used to treat melasma is kojic acid, which acts as a tyrosinase inhibitor through copper chelation. In the treatment of melasma, the best results are achieved in combination with other substances, including HQ, to enhance its action [133]. In a double-blind study, Lim et al. showed that 2% kojic acid in combination with 2% HQ and 10% GA showed better effectiveness than combining 2% HQ with 10% GA [121]. Due to its high effectiveness, kojic acid can be used in patients with intolerance to first-line therapy.

Retinoic acid was also used in the form of peeling at a higher concentration than in daily topical treatment. The effects of tretinoin peels were comparable to those using 70% GA [122]. The substance was also used in the form of masks at a high concentration (10%), leading to very good results and significant clinical improvement [141].

To summarize, chemical peels used alone, as well as in combination with local treatment and laser therapy, achieve good therapeutic effects even in the case of resistant melasma and accelerate such effects [176]. However, they should be used with caution, especially in people with a darker complexion, due to possible side effects and post-inflammatory discoloration.

### 3.3. Laser Therapy

Despite the aforementioned traditional treatments, due to the refractory and recurrent nature of melasma, patients often look for alternative therapeutic strategies that provide rapid improvement, such as laser therapy and light-based therapy. These methods accelerate the removal of melanin, but are not directly targeted at the production of melanin itself. Intense pulsed light (IPL), Q-switched low fluency lasers, non-ablative fractional lasers (NAFL) and picosecond lasers are the most commonly used lasers and light-based treatments in the treatment of melasma. Only the above-mentioned methods will be discussed in this paper, due to the expansiveness of the topic of laser and light-based therapy. All these approaches appear to be effective; however, there is a high risk of recurrence over time, and some techniques are associated with an increased risk of post-inflammatory hyper- or hypopigmentation. Therefore, it is extremely important to explain to patients that these methods can only accelerate the removal of melanin, but remain a causative treatment of this disease. The optimal treatment would be one of a multifactorial action, such as a combination therapy in which topical treatment would inhibit the production of melanin and the transfer of melanosomes to keratinocytes and the laser or light-based treatment would accelerate the removal of melanin. It is worth mentioning that laser therapy and light therapy can also be used effectively in other diseases and conditions with hyperpigmentation [177,178,179].

In 1983, Anderson and Parrish [180] described the use of laser therapy in the treatment of skin diseases for the first time. They noticed a relationship between the selective damage of pigmentary structures in the skin in vivo after the emission of appropriately short pulses of selectively absorbed optical radiation. The destruction of specific structures occurs at certain wavelengths of radiation, while sparing the surrounding tissues. Since then, numerous dermatological diseases and cosmetological defects have been successfully treated with light-based and laser.

IPL is made of an arc lamp that emits incoherent light pulses of various wavelengths (from 515 nm to 1200 nm) and additional filters enable selective action on specific chromophores (melanin, hemoglobin). The potential advantage of this method over laser therapy is the use of the wavelength spectrum, which can penetrate different levels of the skin. The pulse lasts milliseconds, which ensures better thermal diffusion, and the chance of PIH related to tissue overheating is minimized [181,182]. However, the selection of the amount of energy should be performed with caution, as excessively strong values stimulate overactive melanocytes and the development of PIH [182]. Wang et al. [183], in a prospective randomized controlled trial, compared 4% HQ and IPL combination therapy with 4% HQ monotherapy. After the end of treatment, the group treated with HQ and IPL showed a decrease in the relative melanin index of 39.8% compared to 11.6% in the control group treated with HQ only (*p* < 0.05); however, in this study, two patients developed PIH and 24.2% of the participants who improved after IPL developed recurrent pigmentation within 24 weeks of treatment. Yi et al. [184] conducted a meta-analysis and showed that IPL-based combination therapy can effectively lower MASI and results in higher patient satisfaction. This is confirmed by numerous studies assessing the effectiveness of IPL with topical treatment or laser treatment, including three-component therapy [185,186,187,188,189]. In conclusion, it appears that IPL therapy may be effective in patients with melasma refractory to topical therapy alone; however, it should be combined with strong topical treatment, including HQ or ternary cream for at least 6 to 12 months after surgery to avoid relapses. IPL therapy is best suited for the treatment of patients with phototype I-III as well as epidermal melasma [182,190].

Q-switched lasers are one of the most widely used lasers in the treatment of melasma. They produce laser beams of high intensity and a very short pulse duration. The pulse rate of the Q-switched laser is approximately one million times faster than that of the IPL pulse. These melanin-targeting lasers are available in many wavelengths, such as ruby (694 nm), alexandrite (755 nm), and neodymium yag (Nd: YAG; 532 nm or 1064 nm). The standard treatment parameters used in the past had adverse effects, based on the action of photothermolysis, causing cell death, damage to cell membranes and nuclei, the release of prostaglandins and destruction of the basement membrane. Furthermore, the treatment was complicated by postoperative discoloration [191,192,193]. Currently, low-fluence Q-switched lasers are mainly used in the treatment of melasma [182,194]. Low-intensity treatments mainly use the 1064 nm wavelength, which penetrates deeper into the dermis and leaves the epidermis relatively intact. The laser toning technique (low-fluence) involves multiple passes with a low fluency and large spot size, and is associated with the destruction of melanosomes and melanin in keratinocytes, while keeping the cell membrane and nucleus intact (subcellular selective photothermolysis), providing a long-lasting effect of hypopigmentation as a result [195,196,197,198]. Since there is no cell death and skin heating is kept to a minimum, the risk of melasma exacerbation is much lower. This technique achieved the best results and the lowest risk of recurrence when it is combined with other agents such as topical HQ [199,200], triple combination cream [201], azelaic acid [202], chemical peels, e.g., Jessner’s formula [203,204], GA [205,206] and systemic treatment with TA [199,207,208,209,210], as well as other treatment procedures, such as microneedling with ascorbic acid [211], microneedle radiofrequency (RF) [212], microdermabrasion [213], pulsed-dye laser [214], and IPL [188,215,216]. In each of these cases, combination therapy was more effective than the laser toning procedure alone [197,217]. It is worth noting that in 2012, the FDA approved the dual-pulse Nd: YAG Spectra laser for the treatment of melasma patients, making it the first and only approved Q-switched laser therapy for the treatment of patients with melasma. However, despite the good therapeutic effects, the recurrence rate after this procedure remains very high [218,219,220]. The substantial disadvantage of this method is the great number of treatments needed to achieve a therapeutic effect compared to other light and laser treatments; moreover, the procedure should be performed even every 7 days. This procedure is not free from complications and remains a further therapeutic line used in the case of the limited effectiveness of other local treatments. It is, in fact, reserved for very resistant cases where other laser and light-based methods have failed [182].

Fractional lasers create columns of thermal microdamages in the skin. Treated areas are intertwined with undamaged zones (fractional resurfacing), which results in faster convalescence and less inflammation. Fractional resurfacing includes NAFL and ablative fractional laser (AFL) [221].

In the case of NAFL, the water molecule contained in tissues is the target, but columns of coagulation damage are formed in the dermis and the stratum corneum remains intact. The most common symptom immediately after the procedure is redness and swelling. The following four NAFL wavelengths are used: 1440 nm, 1540 nm, 1550 nm, and 1927 nm. NAFL at 1440 nm, 1540 nm and 1550 nm uses mid-infrared waves that penetrate from the dermal–epidermal junction to the middle of the reticular layer of the dermis (maximum depth around 1500 microns), which stimulates collagen production and remodeling. The 1927 nm NAFL thulium laser was introduced later than the other NAFLs. The water absorption coefficient of this laser is 10 times higher than the 1440 nm, 1540 nm and 1550 nm lasers; therefore, it penetrates only to a maximum depth of about 200 microns, which is the depth of the dermal–epidermal junction and the superficial layers of the dermis and the main zone positions of melanosomes and melanophages. The use of NAFLs seems to provide a more sustained clinical response than IPL or Q-switched laser treatments, especially when patients use topical tyrosinase inhibitor treatment before and after surgery. Numerous studies show the effectiveness of NAFLs [178,222,223,224,225,226]; although, for all types of NAFL the recurrence of pigmented lesions occurs. The data suggest that relapse is observed between 3 and 6 months, while with IPL and Q-switched, it occurs as early as 3 months after stopping treatment [182]. The 1927 nm wavelength may offer a more effective response to a single treatment compared to all other devices and its main advantage is the ability to treat patients who also have high Fitzpatrick skin phototypes III to VI, compared to IPL and Q-switched lasers that should be used only for lower skin phototypes. AFLs are not recommended in the treatment of melasma due to the large number of side effects and relapses. If specialists use these treatments, they use a CO_2_ laser with a very low fluency and only in combination therapy [227].

Picosecond lasers are state-of-the-art lasers that generate pulses in the picosecond domain. Shorter laser pulse durations cause melanin fragmentation, which is photoacoustic rather than photothermal. This laser is more effective at removing pigment without causing thermal damage to the surrounding tissues. Picosecond lasers are available with heads emitting different lengths of 532 nm, 755 nm and 1064 nm. Due to the potential of picosecond lasers to act through photoacoustic mechanisms, they may represent a new treatment modality suitable for patients with melasma. Despite the sparse research that has been conducted on the subject [228,229,230], picosecond lasers are mentioned by Sarkar et al. as one of the recommended therapeutic options in patients with melasma [227].

It is also relevant to mention microneedle RF, which is not a laser technique and its mechanism of action is to generate an electromagnetic wave with radio frequency, which, in contact with the tissue, encounters impedance, and generates thermal energy and stimulates the production of collagen. This technique has become much more popular in recent years due to its highly promising results in increasing skin tension, firming and stimulating collagen production, and treating photoaging of the skin, as well as its high safety profile and short recovery time after treatment, making it an excellent adjunct to melasma therapy [231,232]. In addition, RF technology is pigment-independent, which means that it can be used safely in patients with all skin phototypes and has a low risk of discoloration unless there is excessive tissue overheating or arc burns on the skin surface due to inappropriate treatment [233]. Microneedle RF treatment can be combined with the topical application of a tyrosinase inhibitor preparation [234]; however, further research is needed before this procedure can be widely used in the treatment of melasma.

### 3.4. Mesotherapy

Mesotherapy is worth mentioning, since this method is widely used in aesthetic and regenerative dermatology; however, scientific reports on its use in the treatment of melasma are limited. Researchers present the results of the use of various substances in the treatment of melasma, including TA, vitamin C, glutathione, or even triamcinolone. There is the most evidence for the use of TA for mesotherapy in this disease, but the use of ascorbic acid or glutathione requires further research.

When TA is used orally, it effectively inhibits melanogenesis. Khalili et al. reviewed the literature for studies on the use of mesotherapy with TA in the therapy of melasma. The authors included 15 randomized controlled trials evaluating the effectiveness of TA mesotherapy. The studies reported a significant decrease in the size of the affected area and MASI. The procedures were performed at intervals of 1 to 6 weeks. Punctures were made to a depth of 1 mm, every 1 cm, using a 28–30 G needle. The frequency of relapses ranged from 0 to 100% in individual studies, but even in the case of relapses, the degree of skin pigmentation was lower than at the baseline [235].

The work of Mumtaz et al. [236] is also of interest. They compared the effectiveness of mesotherapy with TA and platelet-rich plasma (PRP). According to the researchers, PRP showed better results than TA after 4, 12 and 14 weeks. Therefore, they argued that PPR is more effective than TA in the long-term treatment of melasma with mesotherapy. Additionally, Sarkar and Gupta [237], in a systematic review, presented mesotherapy with PRP as an effective agent in the treatment of melasma, inhibiting melanin synthesis in a multi-level and multi-directional manner.

According to the researchers, PRP can also be used with microneedling with good results. They present PRP as an effective method of treating melasma, even in monotherapy. Growth factors contained in PRP, e.g., TGF-β, reduce the amount of tyrosinase and TRP and also have a positive effect on collagen synthesis, skin quality and texture, as well as reverse photoaging processes [238,239]. However, they emphasize the need to conduct large randomized controlled trials in this direction so that this procedure can become part of everyday clinical practice in the treatment of melasma.

It is also worth emphasizing the role of microneedling, which when combined with active substances, e.g., tyrosinase inhibitors, TA, vitamin C, PRP seems to be an effective and safe additional and complementary treatment method, with a relatively low relapse rate, and can be used in daily medical practice [240,241,242].

### 3.5. Prevention

Photoprotection is fundamental for the treatment and control of melasma. Regardless of the choice of treatment method, sun protection is crucial in preventing the formation of new hyperpigmentation lesions and the aggravation of existing ones. According to recent reports, visible light, especially high-energy visible light (HEVL) and long-wave UVA (UVA1) radiation, plays a significant role in the pathophysiology of melasma, especially in people with a darker complexion [243,244,245]. Photoprotection is equally effective regardless of the type of complexion; however, patients with darker skin tend to use fewer photoprotective measures. Additionally, a large percentage of patients do not adhere to the recommendations for photoprotection, including the proper application of sunscreens [243].

Experts recommend the use of a broad-spectrum UVA/UVB sunscreen with a high sun protection factor (≥SPF 30+) and high protection against UVA1 and HEVL. This requires the use of inorganic filters with a broad spectrum of action with zinc oxide and titanium dioxide and filters colored with iron compounds, which provide protection against HEVL and UVA1 [245,246,247]. Sunscreens for melasma should contain other substances that help treat this skin condition (e.g., antioxidants, anti-inflammatory agents, immunomodulators) or depigmenting agents that improve response to treatment. A high quality formula improves adherence; therefore, the preparation should be water-based, non-greasy and easy to apply without leaving any white residue on the skin. Tinted sunscreens matching skin tones act as camouflage and increase patient satisfaction [243].

## 4. Conclusions

Treatment plans for patients with melasma usually begin with the management or elimination of risk factors, strict protection against ultraviolet radiation, and the topical use of lightening agents. Topical treatments can temporarily improve the condition of the skin, but the problem often recurs. The main principles of the therapy of discoloration in melasma include the inhibition of melanin synthesis pathways, reduction in melanosome transfer from melanocytes to keratinocytes, and acceleration of melanin removal pathways. The process of skin photoaging and excessive neovascularization also seem to be extremely important; therefore, the treatment of hyperpigmentation changes alone will not lead to the expected benefits. Rather, it should be combined with effective regenerative methods and effective protection against light. The appropriate discussions and the establishment of a relationship with the patient seem to be key in the therapeutic process to achieve good adherence and compliance in this long-term, time-consuming and difficult procedure.

## Data Availability

Not applicable.
